# Divergent pathways in urban residents’ emergency behavior in China: a social cognition theory perspective based on a survey of 6,817 individuals across 72 communities

**DOI:** 10.3389/fpubh.2025.1621114

**Published:** 2025-06-19

**Authors:** Chao Wang, Haocun Zhao, Yijue Zhang, Ruyi Shi

**Affiliations:** School of Public Policy and Management, China University of Mining and Technology, Xuzhou, China

**Keywords:** community disaster risk reduction, social cognition theory, behavior motivation, emergency behavior, disaster experience

## Abstract

**Introduction:**

For communities to effectively reduce disasters, the mobilization and guidance of urban residents’ emergency behavior are essential. Community disaster reduction efforts can become more targeted and accurate when the different influencing factors behind different types of emergency behavior are clearly understood.

**Methods:**

We classify emergency behavior into two categories—self-help and mutual aid—based on differences in residents’ behavioral motivations. A coupled “cognition-environment-behavior” driving model has been constructed, drawing upon social cognition theory, to study the mechanisms that drive residents’ emergency actions. The research empirically analyzes factors influencing residents’ emergency behavior during community disasters, utilizing a sample dataset from 72 communities across China that included 6,817 participants.

**Results:**

Three findings are obtained from this study. (1) The extent of residents’ emergency knowledge and skills, with the public dissemination of community information, significantly affects the adoption of self-help emergency behavior. (2) Emergency emotional states, alongside community cultural propaganda, tend to promote mutual aid emergency behavior. (3) Experience with disasters significantly moderates how the community’s disaster mitigation environment affects residents’ emergency behavior.

**Discussion:**

This study not only emphasize key differences in factors across various types of resident behavior but also offer theoretical direction and practical points of reference for enabling targeted responses in community disaster mitigation.

## 1 Introduction

In recent years, sudden public safety incidents have become increasingly frequent, posing significant threats to human lives and properties, and having far-reaching impacts on economic development and social stability. The disaster loss and statistical data from the United Nations Office for Disaster Risk Reduction (UNDRR) show that in 2024, 393 natural hazard-related disaster events occurred worldwide, resulting in $32 billion in losses, 16,753 deaths, and an affected population of over 167 million people. The importance of disaster prevention and response is becoming increasingly prominent. The community acts as the primary defense in disaster prevention, reduction, and relief activities, and it stands at the forefront of disaster management. In China’s disaster risk management, the government has actively encouraged residents’ actions in disaster prevention and emergency response. The Third Plenary Session of the 20th CPC Central Committee emphasized that strengthening grassroots emergency preparedness and capacities is inseparable from broad resident participation. For 2024, the National Disaster Reduction Day theme was established as “Everyone Talks about Safety, Everyone Learns to Respond Safely—Focusing on Enhancing Grassroots Residents’ Disaster-avoidance and Risk-reduction Abilities.” This theme points to dual objectives: improving residents’ emergency literacy and optimizing collective actions for disaster preparedness. Then, numerous local disaster reduction and mitigation plans have stressed the importance of strengthening safety education in both communities and households. Such efforts are designed to enhance residents’ self-help skills and their understanding of mutual aid. This focus highlights how crucial it is to guide residents toward forming standardized and proactive emergency behaviors, as this is critical for improving grassroots community disaster risk reduction capabilities.

In situations requiring disaster response, balancing community residents’ emergency behaviors for disaster prevention and response is vitally important. Consider the severe flood disaster in Zhengzhou, China, in July 2021 for instance, the extreme weather event triggered urban flooding, traffic gridlock, and damaged infrastructure with heavy casualties and property losses. It should be acknowledged that a portion of the residents, influenced by cognitive biases and their limited self-help abilities, demonstrated an excessive reliance on cooperative mutual aid. Such reliance resulted in wasted resources and increased time costs. Moreover, in the absence of community emergency collaborative mechanisms, individual isolated self-help behaviors led to information silos and a dispersion of responsibilities. While grassroots disaster prevention and reduction efforts have achieved some success in recent years, a challenge remains in appropriately guiding residents’ emergency actions to effectively balance individual self-help strategies with mutual aid (1). In practice, the logic behind individual behavior in emergency scenarios often demonstrates significant differences and complexities. For instance, in emergency evacuation scenarios, individuals display different decision-making logics when choosing between departing or cooperating, depending on varying crowd densities and other external environmental factors (2).

Hence, several questions are posed: Does the heterogeneity of individual emergency behaviors during sudden public crises reflect differences in the interconnected elements that drive these behaviors? In what ways do individual cognitive drivers and community environmental drivers influence and act upon individual emergency behaviors? Are there differences in the key factors that affect different types of emergency actions? Do the core driving factors differ structurally among various demographic groups? In light of these questions, this study seeks to enhance residents’ emergency capabilities. This will be achieved by analyzing the heterogeneity of factors that affect different types of emergency behaviors. The research constructs a coupled “cognition-environment-behavior” model and offers targeted policy recommendations to improve residents’ emergency abilities, thereby offering practical guidance for residents’ involvement in disaster reduction efforts.

## 2 Literature review

Concepts, classifications, and determinants are central to the study of emergency behavior. Researchers explore the categorization of public emergency behavior utilizing multiple dimensions, which include content and expression. One perspective systematically classifies public emergency behaviors into three dimensions based on response stages: Emergency reaction, Emergency handling, and Emergency completion (3). Individuals’ information security risk management behavior has also been categorized by studies, distinguishing between the adoption of security technology and security-conscious care behavior related to computer and Internet usage (4). In addition, the analysis of specific scenarios, such as chemical industrial parks, involves dividing their safety emergency behaviors into initial response, field rescue, and post-disaster reconstruction phases (5). Zhong et al. (6) offer another perspective, classifying emergency behaviors from two aspects based on behavior expression forms: Individual Cognition and Social Evaluation. Meanwhile, a differentiation between Compliant and Participatory Safety Behaviors is made by some scholars (7). These categories are further segmented by other studies into different levels, for instance, “The Self–Work–Home–Industry/Society” systems (8).

Public emergency behavior’s driving factors exhibit multi-layered and multi-dimensional characteristics. Current research primarily concentrates on factors that are individual-driven and environmental-driven. The individual level indicates complexity in areas such as awareness, capability, and motivation. In the awareness dimension, research demonstrates that residents’ willingness to participate in community disaster reduction is significantly affected by collective consciousness and individual sense of responsibility (9). Secondly, the capability dimension includes prior experience (10), knowledge stock, emergency knowledge education (11), and emotional control capacity (12), all of which closely relate to residents’ willingness. Maintaining the stability and reasonableness of decision-making is aided by positive emotional control; conversely, negative emotions often result in more conservative behavior decisions (13). Finally, key drivers for public participation in community disaster reduction, in the motivation dimension, are considered to be motives for balancing interests and motives for fairness and reciprocity (14). The protective motivation theory suggests that protective motives and threat assessments influence residents’ behavior tendencies (15).

From the environmental perspective, multiple factors, including informational, social, and physical environments, influence community residents’ emergency behavior. First, cognitive resources are a prerequisite for triggering emergency behavior at the information level. Specific behaviors can result from an individual’s selective processing and cognition of the external environment and adjacent nodes (16). Decision-making, however, can be affected by information irrelevance or overloading (17, 18). Second, in hazard risk scenarios, the social perspective demonstrates that cooperation between individuals and multiple stakeholders is enhanced by richer communication channels in close social networks and by accumulated social capital (19). The prominence of social capital in residents’ learning and participation in community emergency management is indicated by research (20). Finally, an individual’s behavior is significantly affected by their environmental perception at the physical level (21). Studies demonstrate that key factors affecting residents’ participation in community emergency preparedness include safe home environments, resource availability, and incentive systems (10).

The preceding analysis indicates that existing studies focus primarily on normal governance scenarios. These studies often lack a systematic exploration of individual behavior during emergency periods. Besides, gaps also exist in the influencing factors analyses as current research primarily relies on single-dimensional analysis models, thereby not sufficiently considering the complex dynamic interactions between individuals and their environments. In addition, classification of public emergency behavior typically relies on external characteristics such as specific content or forms, while examinations of individual internal motivation categories or their influence mechanisms on specific emergency behaviors are rare. For effective community disaster prevention and reduction efforts, a foundation of support from residents’ corresponding emergency behaviors is required. This study offers several marginal contributions. (1) It categorizes residents’ emergency behavior into self-help and mutual aid from the perspective of behavioral motivation differences, with the aim of establishing a scientific understanding of these behavior types. (2) An integrated driving model for residents’ emergency behavior is constructed, based on grassroots disaster prevention and mitigation scenarios; this model indicates the influencing effects of cognition and environment on behavior. (3) The study explores differential driving factors for various types of emergency behaviors and evaluates the moderating effects of disaster experience on the relationships among cognition, environment, and behavior. Effective guidance for the targeted promotion of residents’ emergency behavior will be offered by the study’s results.

## 3 Theoretical foundation and hypotheses

Bandura’s and Cliffs (22) social cognition theory of human behaviors offers the theoretical basis for this study. This theory hypothesizes that the effect of both internal cognition and the external environment results in individual behavior (22). A study of how individuals’ cognition and social environment influence behavior is central to this theory, and these interactions form an interrelated network represented by “environment-driven, cognition-guided, and behavior-shaped” dynamics (23).

In sudden disaster scenarios, multifaceted environmental factors significantly affect residents’ emergency behavior choices. A “Social-Information-Physical” three-dimensional spatial framework grounds this study (24), which explores the specific impacts of objective environmental conditions on residents’ emergency behaviors, aiming to investigate how objective environmental conditions specifically impact residents’ emergency behaviors. Residents’ willingness to engage in emergency behavior is influenced by social relationship networks, cultural propaganda, and value shaping in the social space (25). In the information space, effective support for residents taking more proactive emergency actions can be offered by high transparency and accuracy of information resources (26). Improvements in safety infrastructure configurations allow physical space factors to directly influence residents’ behavioral decision preferences (27). Therefore, this study selects cultural propaganda (CP), information publicization (IP), and basic emergency response infrastructure (BRI) as key community disaster mitigation environment factors.

An individual’s knowledge and beliefs, formed through interaction with the objective world, define their cognitive structure; this formation results from the integration of subjective perceptions of reality with existing knowledge and experiences (28). Three core dimensions are the focus of this study: metacognitive awareness, emotional understanding, and knowledge structure. The effect of residents’ cognitive appraisal and approach to a task on their performance, through integration mechanisms, is emphasized by metacognitive awareness theory (29). The individual’s deep cognitive processes concerning their own emotions and those of others constitute emotional understanding, and these processes regulate behavior selection (30). Knowledge structure defines the ability of individuals to acquire, assess, and apply knowledge in decision-making (31). The cognitive foundation that affects emergency behavior is collectively formed by these cognitive dimensions. Therefore, individual emergency cognition factors selected for this study include emergency awareness (EA), emergency emotion (EM), and emergency knowledge and skills (EKS).

Based on scenario characteristics, actor elements, and the lifecycle of incident features (6), this study defines residents’ emergency behavior as: a set of integrated, preventive, and adaptive actions taken by residents during an incident to reduce damage and ensure their own and others’ safety (32). From the perspective of behavioral motivation differences (33), community residents’ emergency behavior is categorized by this study into two types: self-help and mutual aid (Table 1). The former is motivated by individual autonomous assessment of self-needs, emphasizing the use of individual ability and resources. It focuses on individual-level safety guarantees, such as family disaster preparedness behaviors and information-seeking behaviors. The latter is driven by community group collaboration, highlighting resource complementation and mutual assistance at the collective level (11). It concentrates on group-level overall safety assurance, such as mutual-rescue behaviors and participation in community emergency drills.

**Table 1:** Classification of community residents’ emergency behavior based on motivation differences.

ClassificationMotivation differences	Self-help emergency behavior	Mutual aid emergency behavior
Motivation	Self-interest; self-protection	Benevolence expectations
Characteristics	Independence and autonomy	Collaborative and social orientations
Examples	Disaster preparedness behavior; Information-seeking behavior	Mutual-rescue behaviors; Participation in emergency drills

### 3.1 Community disaster mitigation environment and emergency behaviors

Cultural propaganda’s purpose involves advancing educational activities. These activities are designed to update residents’ awareness, beliefs, and behavioral patterns concerning disaster prevention and mitigation, thereby cultivating spontaneous emergency response actions in the populace. Previous research has identified education, behavioral demonstration, and social support as critical strategies that effectively influence the emergency behavior of residents (34). Academically, education has the potential to strengthen both human capital and social capital; in fact, studies suggest a correlation where higher educational attainment is associated with increased engagement in collective activities (35). In addition, knowledge dissemination, the enhancement of individual moral and cognitive capabilities, and the cultivation of compliance with social norms can be achieved through educational interventions (36). These collective insights illustrate the capacity of cultural propaganda to elevate residents’ emergency awareness. It can also impart practical safety knowledge and skills, strengthen community cohesion, and finally equip individuals to implement effective emergency measures when crises occur.

The concept of an open information environment refers to the capacity of community systems or information flows. This capacity involves the reliable and effective transmission of accurate information, even when facing external disturbances, natural disasters, or other challenges (37). Findings from research studies exhibit that a significant rise in media coverage frequency often aligns with a corresponding reduction in disease incidence. For instance, if the volume of reporting multiplies tenfold, the quantity of disease infections can decline by one-third (38). The dissemination of disaster-related information, therefore, significantly affects public behavior and the decision-making processes regarding potential risks (39).

Essential elements that guarantee the stability and resilience of organizational systems in a community define basic community emergency infrastructure; such infrastructure enables organizations to mount effective responses during emergencies. Various factors, as indicated by research findings, exhibit a positive correlation with community resilience, including technical variables, organizational variables, management variables, and prevailing community environmental conditions. More specifically, improvements in community resilience are advanced by technical enhancement, robust organizational construction, effective management planning capabilities, and overall environmental stability (40). Moreover, communities are empowered to make superior decisions and react more swiftly to emergencies through the establishment of strong infrastructure designed to address natural and man-made disasters, coupled with the ongoing maintenance and updating of these critical systems (41). The improvement of community environments, therefore, proves beneficial for enhancing residents’ behaviors that are oriented towards emergency response.

Based upon the preceding analysis, it can be understood that scientific-educational strategies, a consistently stable information public environment, alongside well-established emergency infrastructure and organizational systems, are capable of enhancing residents’ emergency awareness and their self-help capabilities. These enhancements offer crucial support for their effective actions during emergency situations. Therefore, this study proposes the following research hypotheses:

*H1a:* Cultural propaganda positively affects residents’ emergency behavior;

*H1b:* Information publicization positively affects residents’ emergency behavior;

*H1c:* Basic emergency response infrastructure positively affects residents’ emergency behavior.

### 3.2 Individual emergency cognition and emergency behaviors

A close relationship is present between the emergency awareness of residents and their emergency behavior. In this study, emergency awareness signifies an individual’s particular understanding of their physical sensations, psychological experiences, and cognitive activities when in a disaster situation, concurrent with their perception of external stimuli associated with disasters (42). Both this awareness of disaster risks and the awareness of appropriate disaster responses significantly shape residents’ involvement in activities focused on disaster reduction and relief (43). Insufficient emergency awareness, as research findings suggest, stands as a primary factor for individuals choosing not to participate in emergency drills (44). Besides, a foundational contribution to the formation of residents’ behavior comes from a well-established emergency awareness system, while the early cultivation of crisis consciousness tends to promote more consistent long-term participation in emergency training, campaigns, and drills.

Emergency emotion describes a rational emotional state defined by being positive, proactive, and calm. To a certain degree, emergency emotions can prompt the occurrence of emergency behaviors. According to classical high-stakes emotion theory, how individuals behave in emergencies frequently represents an immediate reaction to these experienced emotions (32). In production safety, employees’ unsafe behaviors are significantly affected by emotions; this effect is, accordingly, moderated by group behavior norms and individual control mechanisms (45). In business operation, the psychological dynamic response mechanism for how individuals react to relevant events, actions, or experiences at work is jointly formed by both cognition and emotion. A unique contribution to the depletion of energy resources is made by both negative emotional events and the regulation of these events, often resulting in fatigue, exhaustion, or negative affect (46). Fatigue or emotional exhaustion also increases the probability of dangerous behaviors (47). Accordingly, this study hypothesizes that maintaining a calm and rational emotional state is indispensable for an effective response to disaster events.

Emergency knowledge and skills reflect the set of knowledge and skills that an individual must have to protect themselves and others when a disaster occurs. Academic communities have yet to arrive at a unified agreement concerning the conceptualization and measurement of residents’ emergency competence. The belief held by some scholars is that residents primarily acquire emergency competence through avenues such as knowledge accumulation, propaganda-based education, and practical skill training, among other methods (48). The degree to which residents are equipped with emergency knowledge and skills has a direct bearing on the effectiveness of their emergency behaviors. During emergencies, individuals with high levels of emergency knowledge alongside expertise in relevant skills demonstrate a greater capacity for the rapid identification of dangers and for rendering assistance to themselves or to others.

The aforementioned analysis evidently indicates that factors including emergency awareness, emotional states experienced during emergencies, and the knowledge and skills relevant to emergencies, can potentially affect an individual’s rational judgment. Resultingly, it affects their emergency behavior. Therefore, three hypotheses, which are according to these identified individual emergency cognitions, are proposed by this study:

*H2a:* Emergency awareness positively affects residents’ emergency behavior;

*H2b:* Emotional states during emergencies positively affects residents’ emergency behavior;

*H2c:* Emergency knowledge and skills positively affects residents’ emergency behavior.

### 3.3 The moderating effects of disaster experiences

Related studies in social psychology exhibit that disaster experiences can alter an individual’s experiential cognition. This change frequently leads to heterogeneous risk perceptions and also has the potential to impact decision-making processes (49). According to this understanding, this research evaluates how disaster experiences moderate the relationship between individuals’ emergency cognition, the community disaster mitigation environment, and emergency behavior.

The moderating effect of disaster experiences indicates itself primarily in three key ways. Firstly, residents’ awareness regarding disaster prevention and mitigation, along with their motivation to act, can be significantly strengthened through such experiences. Typically, disaster experiences noticeably enhance an individual’s sensitivity to risk, prompting them to strengthen their understanding of disaster risks through firsthand encounters (50). Moreover, these experiences may genuinely spur psychological transformation, which boosts the drive to aid other vulnerable community members and to participate more proactively in disaster prevention and mitigation efforts (51). Disaster experiences also equip individuals with the ability to accumulate adaptive experience for emergency situations, thereby lessening the negative emotional effects on their actions (52). Secondly, disasters present opportunities for residents to forge connections and strengthen community resilience. The shared empathetic experience during a disaster, alongside a sense of “common sentiment” that cultivates social cohesion rooted in a collective fate, can cultivate deeper interpersonal relationships and encourage mutual support when disasters strike (53). Finally, through the accumulation of personal encounters, disaster experiences can enable residents to develop local knowledge, empowering them to identify and address disaster risks more effectively (54). Individuals can progressively establish a stable reserve of emergency-related knowledge by engaging in reconstruction activities throughout the disaster recovery period (55). This analysis leads the study to put forth the following hypotheses:

*H3a:* Disaster experiences have a positive moderating effect on the relationship between individual emergency cognition and residents’ emergency behavior;

*H3b:* Disaster experiences have a positive moderating effect on the relationship between community disaster mitigation environment and residents’ emergency behavior.

**Figure 1:**
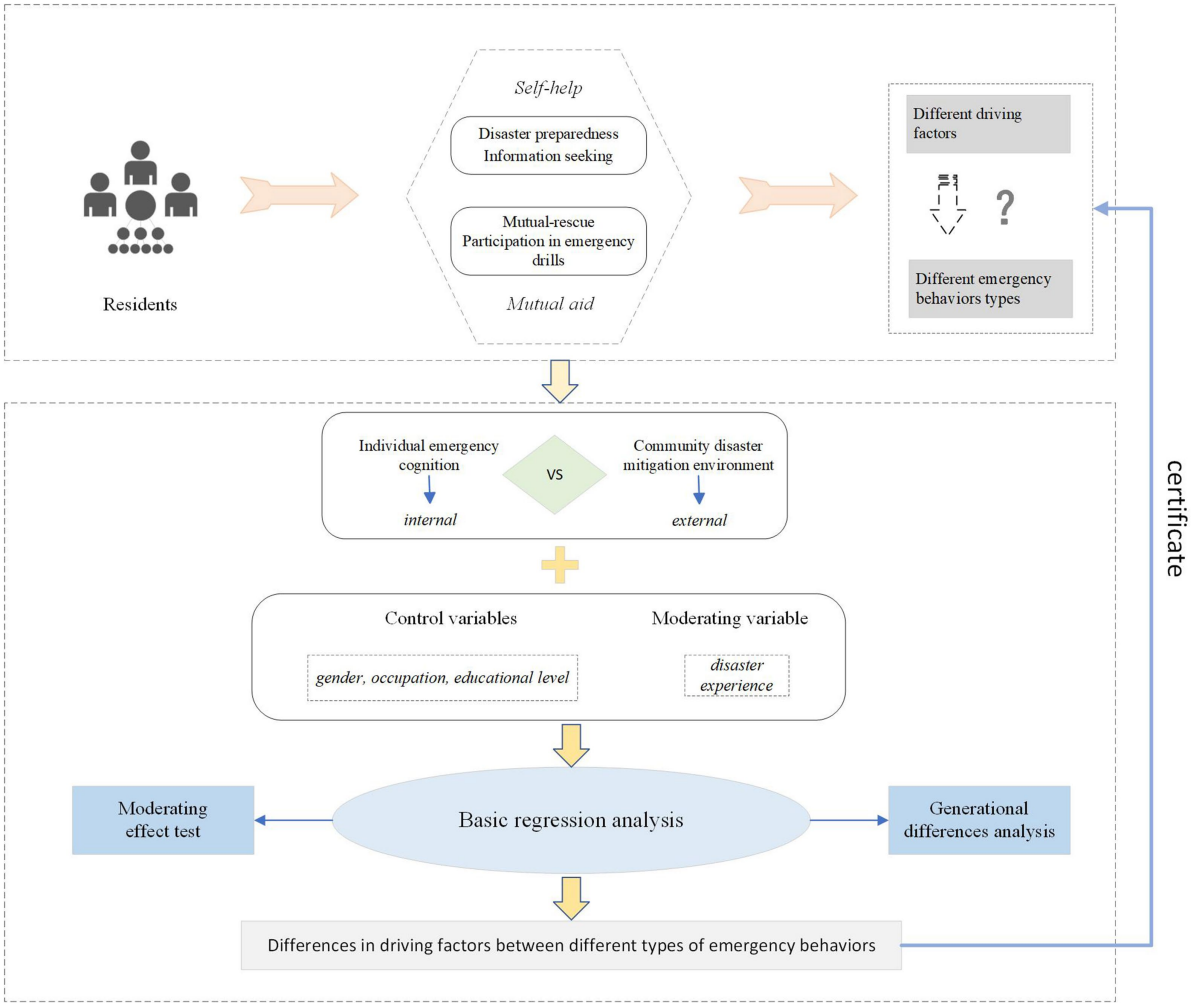
Research framework.

Consequently, using the analyses above as a basis, we created the research framework shown in Figure 1.

## 4 Methods

### 4.1 Model building

This study proceeds to appraise the ways community disaster mitigation environments and individual emergency cognition affect the emergency behavior of residents. To fulfill this objective, the following metric models were developed:

(1) $${\mathrm{SHT}}_{i}=\alpha_{1}+\beta_{1}{\mathrm{CME}}_{i}+\gamma_{1}{\mathrm{ICE}}_{i}+\delta_{1}{\text{control}}_{i}+\varepsilon_{i}$$

(2) $${\mathrm{MAT}}_{i}=\alpha_{2}+\beta_{2}{\mathrm{CME}}_{i}+\gamma_{2}{\mathrm{ICE}}_{i}+\delta_{2}{\text{control}}_{i}+\varepsilon_{i}$$

where ${\mathrm{SHT}}_{i}$ and ${\mathrm{MAT}}_{i}$ represent the self-help and mutual aid emergency behaviors exhibited by the i^th^ resident; ${\mathrm{CME}}_{i}$ denotes the community disaster mitigation environment perceived by the i^th^ resident; ${\mathrm{ICE}}_{i}$ refers to the individual emergency cognition of the i^th^ resident; ${\text{control}}_{i}$ includes control variables such as gender, occupation, educational level, etc., for the i^th^ resident; $\varepsilon_{i}$ expresses the random error term.

This study further analyzes the moderating effects of disaster experiences on the relationships between community disaster mitigation environments and individual emergency cognition with residents’ emergency behavior. Therefore, the following interactive regression model is derived:

(3) $$\begin{array}{l} {\mathrm{EB}}_{i}=\alpha_{3}+\beta_{3}{\mathrm{CME}}_{i}+\gamma_{3}{\mathrm{ICE}}_{i}+\varphi_{3}{\mathrm{DE}}_{i}+\sigma_{3}{\mathrm{CME}}_{i}\\ \times {\mathrm{DE}}_{i}+\omega_{3}{\mathrm{ICE}}_{i}\times {\mathrm{DE}}_{i}+\delta_{3}{\text{control}}_{i}+\varepsilon_{i} \end{array}$$

where ${\mathrm{EB}}_{i}$ refers to the individual emergency behavior of the i^th^ resident; ${\mathrm{DE}}_{i}$ denotes the disaster experience of the i^th^ resident; ${\mathrm{CME}}_{i}\times {\mathrm{DE}}_{i}$ and ${\mathrm{ICE}}_{i}\times {\mathrm{DE}}_{i}$ represent the interactive terms between community disaster mitigation environment and disaster experience, and between individual emergency cognition and disaster experience, respectively. These interactions are employed specifically to test the moderating effects of disaster experiences on such relationships.

### 4.2 Survey design

This study involves the design of a survey instrument using scales. It measures the dependent and independent variables through a five-point Likert scale (1 meant “highly disagree”, 2 meant “disagree”, 3 meant “neutral”, 4 meant “relative agree” and 5 meant “highly agree”) and measures the moderating variable through a summated rating scale (1 meant “yes”, 2 meant “no”). And the content of the survey is organized into two sections. The first section collects fundamental information from participants, including demographic indicators associated with population statistics. For the second section, the items are initially designed by incorporating relevant existing research on variables, and are finally developed by refining them according to the opinions of emergency experts and scholars. Here, the scale for individual emergency cognition covers dimensions such as emergency awareness (42), emergency emotion (56), emergency knowledge and skills (57); higher scores obtained on this scale reflect a greater level of residents’ emergency cognitive capabilities. Internal consistency reliability figures (Cronbach’s alpha coefficients) for the overall scale and its sub-scales are 0.810, 0.798, 0.753, and 0.820, respectively. The community disaster mitigation environment structured around three dimensions: basic emergency response infrastructure (58), information publicization (59), and cultural propaganda (60), with higher scores indicating a higher level of community disaster reduction environmental support. For this total scale and its sub-scales, the internal consistency reliability values are 0.899, 0.830, 0.793, and 0.842, respectively.

The self-help emergency behavior includes information-seeking behavior (61) and disaster preparedness behavior (62), with internal consistency reliability of 0.819, 0.799, and 0.790 for the total scale and sub-scales, respectively. The mutual aid emergency behavior consists of mutual rescue behavior (63) and participation in community emergency drills (64), with internal consistency reliability of 0.741, 0.821, and 0.840, respectively. The higher the score, the higher the corresponding behavioral level of individuals. Whether the community had encountered any sudden disaster events in the 2 years prior is the indicator for disaster experience (65). The reliability and the credibility of the questionnaires satisfied the criteria: the total Cronbach’s alpha of the data was 0.913, and the Cronbach’s alpha value of each question item exceeded 0.7. Strong questionnaire reliability was indicated by the KMO value (0.943) and the *p*-value, which suggested significance at the 1% level. Table 2 presents the definitions and statistical descriptions for all variables and indicators.

**Table 2:** Measurement of specific variables and indicators.

Variable	Definition and measurement	Mean	SD
Emergency awareness			
EA1	When entering public places, I always pay attention to fire protection facilities, emergency exits, escape route maps, and so on.	3.78	1.222
EA2	I am highly alert to unexpected incidents and always consider safety issues first when doing anything.	3.90	1.150
EA3	I attach great importance to the accumulation of safety knowledge.	3.89	1.115
Emergency emotion			
EM1	After detecting abnormal and dangerous situations, I am able to make quick judgments and handle them promptly.	3.66	1.129
EM2	I have strong resistance and can adapt to the effects of various environmental changes and physiological stimuli on my body.	3.59	1.148
EM3	When my emotions fluctuate greatly, I can quickly adjust my emotions to a calm state.	3.73	1.111
Emergency knowledge and skills			
EKS1	I am familiar with various warning signs and can make reasonable use of warning signs.	3.78	1.182
EKS2	I can use emergency rescue facilities such as fire extinguishers and escape ropes.	3.83	1.118
EKS3	I have mastered the basic knowledge of emergency escape in the face of sudden incidents such as floods, fires, and earthquakes.	3.31	1.344
EKS4	I have mastered first aid methods such as cardiopulmonary resuscitation, fracture fixation, wound hemostasis, and bandaging.	3.69	1.160
Basic emergency response infrastructure			
BRI1	The community has comprehensively implemented grid-based management of risks and hidden dangers of disaster accidents.	4.00	1.131
BRI2	The community is equipped with emergency supplies storage points or miniature fire stations.	4.01	1.094
BRI3	The community has a volunteer team that participates in daily comprehensive disaster reduction work.	3.97	1.187
Information publicization			
IP1	The streets and the government release relevant information about emergencies and disasters in a timely and complete manner.	4.04	1.038
IP2	The government has a high level of transparency in disclosing emergency rescue information.	4.05	1.049
IP3	The dissemination of accident and disaster information in the community is rapid and authentic.	3.96	1.086
Cultural propaganda			
CP1	The community will organize activities in various forms to carry out popular science publicity and education on disaster prevention and mitigation.	3.97	1.144
CP2	The community often reminds residents to pay attention to potential safety hazards through loudspeakers, electronic screens and other means.	3.91	1.159
CP3	The community will regularly conduct various emergency safety trainings and distribute family emergency guidance manuals.	3.80	1.225
Self-help emergency behavior			
SHT1	I will learn about accident and disaster information through channels such as private WeChat groups, relatives and friends.	3.75	1.216
SHT2	I am aware of the policies or regulations issued by the community for dealing with various unexpected incidents.	3.72	1.184
SHT3	I often pay attention to the information about unexpected accident disasters on the community APP, government websites, and community bulletin boards.	3.88	1.160
SHT4	I will prepare sufficient emergency supplies, such as food, water sources, first aid supplies, etc.	3.71	1.210
SHT5	I will prepare emergency communication equipment so that I can obtain information in a timely manner in case of an emergency.	3.54	1.296
SHT6	I will prepare basic personal protective equipment, such as masks, gloves, a medical kit, etc.	3.93	1.152
Mutual aid emergency behavior			
MAT1	In daily life, if others encounter unexpected incidents, I will take the initiative to offer help.	4.19	0.961
MAT2	In an emergency, I will provide help within my ability to neighbors in need.	4.25	0.935
MAT3	I will actively participate in the emergency drills regularly organized by the community.	3.73	1.262
MAT4	I will improve my ability to deal with emergency situations by participating in community emergency drills.	3.88	1.210
Disaster experience			
DE	Whether the community has experienced sudden disaster events in the past two years?	1.57	0.495
Control variables			
Gender	Gender (male = 1, female = 0)	1.54	0.499
Occupation	Occupation (civil servant = 1, staff of public institutions = 2, enterprise employee = 3, freelance individual = 4, unemployed = 5, retired individual = 6, student = 7, others = 8)	4.87	2.235
Educational level	Educational level (junior school or less = 1, senior high school = 2, university degree = 3, master’s degree = 4, doctoral degree = 5)	2.46	0.886
Age	Age (18–29 = 1, 30–44 = 2, 45–59 = 3, 60 and above = 4)	2.29	1.025

Highly disagree = 1, disagree = 2, neutral = 3, relative agree = 4, highly agree = 5, yes = 1, no = 2.

### 4.3 Data sources

The data underpinning this study were collected through a survey. This survey was administered by China University of Mining and Technology in July 2024 and was a component of the “Hundreds of Communities Survey” focused on emergency management in China. Considering the population scale, economic development level, and practical operability of survey accessibility factors, this study selected 27 provincial capital cities, 4 municipalities, and 5 special planned cities to conduct the investigation. Provincial capital cities and municipalities are representative and comprehensive, covering all provincial administrative units in the Chinese mainland, while directly reflecting the implementation effects of emergency policies in each province. Additionally, special planned cities, due to their special economic characteristics and the background of reform pilot projects, may exhibit distinct emergency behaviors among residents that deserve special attention. The representativeness and coverage of the data ensure the national applicability and broad relevance of the research results. The offline survey adopted a multi-stage random sampling method, four subdistricts were randomly selected in each city, and two communities were randomly selected in each subdistrict.

During the process of offline community surveys, investigators obtained a list of long-term residents in the community from local community leaders. For each list, 25 households were randomly selected, and one person was chosen from each household as the subject for face-to-face questionnaire surveys. This approach ensured the collection of the most authentic thoughts from respondents and eliminated the influence of tourists and other non-local residents on the survey results. Additionally, all sample data collection was conducted under the supervision of investigators to ensure interviews were completed and returned with high accuracy and quality. A total of 7,127 questionnaires were collected in this survey. After excluding questionnaires that were incomplete or incorrectly filled out, 6,817 valid questionnaires remained, which signifies a response rate of 95.65%. Participants had been provided with clear and comprehensive information about the purpose of our study, the nature of the questions, and how their responses would be used. It is important to note that, due to privacy considerations, the study did not collect data concerning individuals’ mental health or other substance use. All participants’ responses had been treated as confidential and would not be disclosed to third parties.

## 5 Results

### 5.1 Descriptive statistics

Our sample analysis sourced data from the valid questionnaires. Table 3 presents that male and female respondents were represented in nearly equal numbers. When analyzing educational backgrounds, individuals with university qualifications comprised the largest segment, accounting for 48.58%; the sample also included participants from various other educational tiers. The occupational data indicated that enterprise employees, retired persons, civil servants, and freelance workers formed a relatively large proportion. Meanwhile, the respondent pool comprised a range of other roles, including different civil service positions, unemployed individuals, and students. Regarding age distribution, the 30–44 age bracket was the most numerous at 33.77%, whereas the smallest cohort consisted of older adult individuals (60 years and older) at 15.71% of the sample.

**Table 3:** Description of the distribution of sample characteristics.

Characteristic	Frequency	Percentage
Gender		
Male	3,153	46.25%
Female	3,664	53.75%
Education		
Civil servant	247	3.62%
Staff of public institutions	784	11.50%
Enterprise employee	1,426	20.92%
Freelance individual	1,162	17.05%
Unemployed	191	2.80%
Retired individual	913	13.39%
Student	697	10.22%
Others	1,397	20.49%
Education		
Junior school or less	1,169	17.15%
Senior high school	1890	27.72%
University degree	3,312	48.58%
Master’s degree	340	4.99%
Doctoral degree	106	1.55%
Age		
18–29	1810	26.55%
30–44	2,302	33.77%
45–59	1,634	23.97%
60 and above	1,071	15.71%

**Figure 2:**
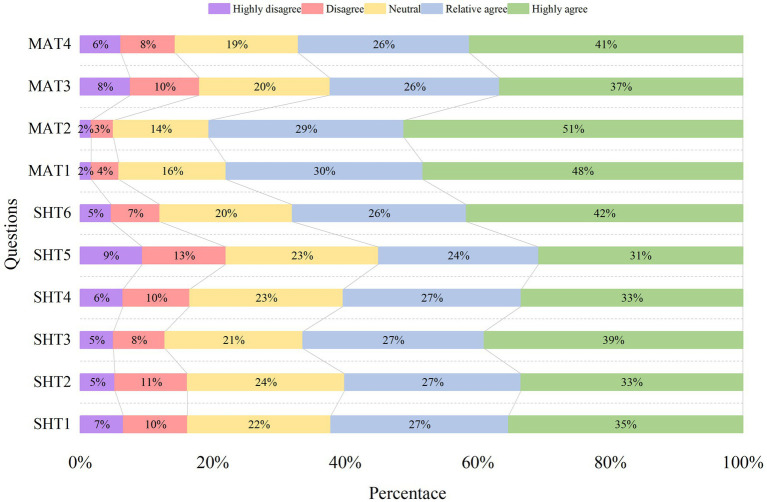
Behavioral decision-making data in the results of the questionnaire.

Statistical methods were employed to analyze questionnaires responses. Figure 2 presents the findings related to behavioral decision-making. These results exhibit penetration rates exceeding 53% for both categories of emergency behavior, indicating significant behavioral tendencies toward these activities. In comparison, residents demonstrated a low willingness for community-organized emergency drills.

### 5.2 Basic regression analysis

The baseline regression results on the effects of individual emergency cognition and the community disaster mitigation environment on two types of emergency behavior, are presented in Table 4. Control variables were not incorporated into models M1, M2, and M4. The findings indicate a significant positive effect of both individual emergency cognition and the community disaster mitigation environment on the emergency behaviors of residents, thereby confirming hypotheses H1a, H1b, H1c, H2a, H2b, and H2c. To account for potential omitted variable bias, models M3 and M5 were developed with the inclusion of control variables. The analysis demonstrated that the influence coefficients of all observed variables on residents’ emergency behavior remained significantly positive, and their magnitudes changed minimally, further emphasizing the robustness of this study’s conclusions.

**Table 4:** The regression results of the basic model.

Variable	EB	Self-help		Mutual aid	
	M1	M2	M3	M4	M5
EA	0.104***	0.092***	0.091***	0.099***	0.097***
EM	0.151***	0.148***	0.149***	0.122***	0.123***
EKS	0.206***	0.249***	0.251***	0.092***	0.095***
CP	0.242***	0.171***	0.170***	0.299***	0.297***
BRI	0.134***	0.087***	0.086***	0.177***	0.177***
IP	0.280***	0.290***	0.290***	0.202***	0.202***
Gender			0.012		0.013*
Education			−0.011		−0.005
Occupation			0.007		0.018**
R^2^	0.848	0.788	0.789	0.763	0.763
Adjusted R^2^	0.718	0.621	0.621	0.582	0.582

*p < 0.10, **p < 0.05, ***p < 0.01.

When considering self-help emergency behavior, the presence of emergency knowledge and skills (in individual emergency cognition) and the public availability of information (in the community disaster mitigation environment) appear to be more crucial factors for residents adopting self-protective measures. This phenomenon might occur as residents, through processes of learning and practice, assimilate emergency knowledge and skills to the point where they become habitual; these ingrained habits then unconsciously guide individuals toward appropriate emergency responses, often without their explicit awareness. From a behavioral decision-making perspective, an increase in both the scope and proficiency of knowledge and skills builds greater confidence among residents. This greater confidence, accordingly, results in increased self-effectiveness concerning their intended behaviors (66). Besides, a key principle of cognitive science is information processing (67). As information transparency improves, residents tend to place greater trust in information sources. This trust is vital for protecting the communication pathways between organizations and individuals, which are dependent on the flow and processing of information, thereby encouraging behavioral attitude changes.

In mutual aid emergency behavior, emergency emotions (as a component of individual emergency cognition) and cultural propaganda (an aspect of the community disaster mitigation environment) more significantly affect on the collective mutual assistance actions of residents. The emotional and psychological bonds that exist between individuals and the groups they form in a community could explain this pattern. Core characteristics of a community, such as emotional connection and trust, enhance the willingness of residents to engage in mutual aid (68). Moreover, emergency resilience education activities are often implemented at the community level. These activities contribute to strengthening the ties and cohesion among community members, thereby cultivating the development of a community-wide emergency culture. Residents are more disposed to offer support and collaborate in addressing community disaster challenges once they have established robust networks of trust.

### 5.3 Analysis of the moderating effect of disaster experience

This research employed the SPSS macro program PROCESS 4.1 to explore the moderating effect of disaster experience on the connections between individual emergency cognition, the community disaster mitigation environment, and emergency behavior, following the moderation model testing method proposed by Wen et al. (69). An analysis of the moderating effects (Table 5) indicated that disaster experience does not act as a moderator on the relationship between individual emergency cognition and emergency behavior. Accordingly, hypothesis H3a was not supported. One possible reason is the somewhat general method disaster experiences were measured, which did not differentiate their specific nature, severity, or type. It is conceivable that minor disaster experiences are not impactful enough to significantly change an individual’s cognitive processes or behaviors; whereas, severe disaster experiences have the potential to cause significant psychological trauma and stress responses. With the passage of time, individuals may gradually adapt, returning to their usual psychological states and behavioral modes, and this adaptation could reduce the moderating role of disaster experiences on emergency actions. Data from models M9, M10, and M11 demonstrated that disaster experience significantly moderates the relationships involving cultural propaganda, basic emergency response infrastructure, and information publicization with emergency behavior; these findings also satisfied the 1% significance level test. It implies that disaster experience enhances the positive effects of cultural propaganda, basic emergency response infrastructure, and information dissemination on residents’ emergency conduct. Therefore, hypothesis H3b found validation through these results.

**Table 5:** Mechanism test of disaster experiences.

Model	Variable	β
M6	EA	0.511***
	DE	0.092***
	DE*EA	0.005
	control	YES
	R^2^	0.279
M7	EM	0.558***
	DE	0.079***
	DE*EM	−0.006
	control	YES
	R^2^	0.324
M8	EKS	0.625***
	DE	0.082***
	DE*EKS	0.018
	control	YES
	R^2^	0.404
M9	CP	0.681***
	DE	0.065***
	DE*CP	0.030***
	control	YES
	R^2^	0.690
M10	BRI	0.643**
	DE	0.061***
	DE*BRI	0.026***
	control	YES
	R^2^	0.426
M11	IP	0.695***
	DE	0.056***
	DE*IP	0.023***
	control	YES
	R^2^	0.495

*p < 0.10, **p < 0.05, ***p < 0.01.

### 5.4 Generational differences analysis

Considering that older residents frequently contend with issues such as cognitive decline, social impediments, and a reduced sense of effectiveness when reacting to emergencies, this study segmented residents into two age-based classifications: those younger than 60 years and those 60 years or older. The findings presented in Table 6 demonstrate that an understanding of emergency basics significantly increases the likelihood of emergency behavior among the younger demographic (under 60). However, for the older group, the effect of these basics on self-help emergency behavior did not achieve significance. The comparatively entrenched individual behavioral patterns of residents aged 60 and above could account for this difference. Such individuals generally exhibit a strong dependence on past experiences when confronting emergencies and are less inclined to carry out behavioral shifts, even if their knowledge of emergency fundamentals improves. Moreover, limitations related to physical capabilities and fitness levels place further restrictions on their capacity for rapid adjustment to emergency situations. This constraint contributes to the maintenance of their long-established self-help behavioral tendencies during crises (70).

**Table 6:** Regression results of the analysis of generational differences.

Variable	Mutual aid		Self-help	
	Under 60 years old	Over 60 years old	Under 60 years old	Over 60 years old
EKS	0.090***	0.115***	0.240***	0.306***
EA	0.097***	0.103***	0.101***	0.050*
EM	0.116***	0.147***	0.147***	0.144***
CP	0.298***	0.299***	0.170***	0.172***
BRI	0.178***	0.169***	0.097***	0.032
IP	0.209***	0.166***	0.281***	0.334***
R^2^	0.763	0.758	0.785	0.805
Adjusted R^2^	0.582	0.572	0.615	0.674
N	5,746	1,071	5,746	1,071

*p < 0.10, **p < 0.05, ***p < 0.01.

## 6 Discussion

The continuous advancements in public crisis management research have led scholars to increasingly investigate individual behavior during disasters and crises (71). Grassroots communities and units, often called the “last mile” of societal governance, are both central to the daily lives of the population and essential for maintaining public safety. Since various hazards and crises display unique characteristics, different forms of emergency education activities have been progressively incorporated into grassroots emergency management systems; the purpose of this integration is to systematically strengthen residents’ abilities to act as protectors during emergencies. This study, drawing inspiration from Lainas and employing a perspective according to social cognition theory, categorizes residents’ emergency behaviors into self-help and mutual aid to deeply analyze their behavioral patterns under such conditions (72). Through this research, unique insights are offered into how cognitive factors alongside environmental influences shape the characteristics of residents’ emergency behavior.

### 6.1 Residents’ decision-making of emergency behaviors

**Figure 3:**
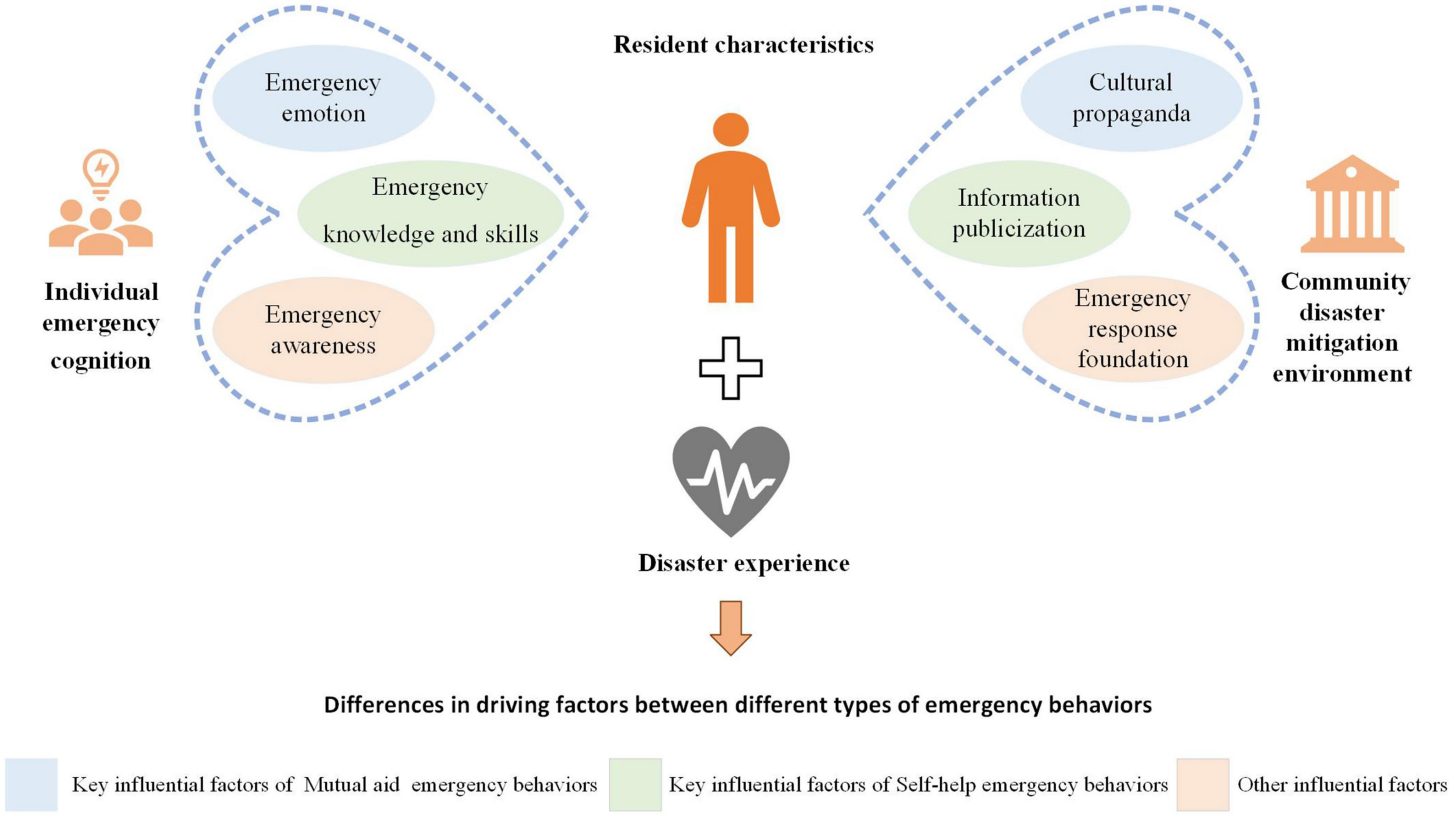
Differences in emergency behavioral decision-making.

Residents’ emergency behaviors are driven by multiple influencing factors. The findings of the study, illustrated in Figure 3, highlight emergency knowledge and skills as a crucial determinant of an individual’s likelihood to engage in self-help behaviors amid crises. This observation aligns with the concept of self-efficacy given by Bandura (73), which suggests that an individual’s belief in their own capabilities strongly affects their confidence in performing self-help actions. Therefore, more comprehensive emergency knowledge and skills appears to boost residents’ self-effectiveness when facing disaster risks, thereby prompting greater initiative in their emergency decision-making during urgent situations. Liu et al. (74) present findings consistent as well, demonstrating that individuals with greater expertise in knowledge and skills related to floods are more inclined to initiate evacuation during such disasters. Moreover, the availability of solid emergency knowledge and skills strengthens residents’ confidence, which promotes more active engagement in emergency response efforts and the implementation of self-help measures. Communities, for this reason, should offer easily accessible resources, such as practical, user-friendly knowledge and skills designed for emergency situations. To facilitate learning and application, they should also render concise operational guides for complex tools. Communities could also offer more flexibility, enabling residents to select courses that align best with their individual needs to improve the effectiveness of skill development programs.

Regarding another key factor that affects self-help emergency behavior, residents’ access to open and transparent information has significantly improved the effectiveness of emergency responses involving their participation. This finding is consistent with several studies indicating that better information acquisition positively affects participants’ engagement levels during emergencies. Griffin’s et al. (75) work, which proposed that information sufficiency, perceived capacity for information collection, and beliefs about relevant channels could influence decisions to take effective action, aligns with this result; whereas, if residents experience challenge obtaining disaster resources, their emergency behaviors might be adversely affected (76). From the viewpoint of social cognition theory, the accessibility of disaster-related information is a fundamental component of environments designed for disaster risk mitigation. It has a direct impact on how residents perceive the effectiveness of emergency measures and the means they engage in emergency actions. By improving the efficiency and transparency of publicly available disaster information in communities, potential barriers residents face during emergencies can be reduced, allowing them to access critical information more readily in times of crisis. It is expected that such a transparent communication strategy will further build residents’ confidence in utilizing self-help strategies during emergencies, which finally strengthens their capacity to act as protectors and contribute to efforts reducing disaster risk.

Emotion regulation stands as a vital cognitive function for adapting to life’s challenges. Research indicates that a positive emotional state contributes to stimulating residents’ motivation for mutual rescue. Hammond’s study supports this observation by emphasizing the importance of positive emotions for prosocial behavior and development (77). Positive emotions, which include empathy and a sense of responsibility, encourage mutual aid behaviors. A higher sense of responsibility compels individuals to regard mutual aid as an indispensable obligation. Moreover, instinctual responses can be further prompted by moderate levels of psychological fear, encouraging individuals to take immediate action during crises, such as seeking assistance from others or offering support to those in need. Effective positive emotional management also assists residents in minimizing defensive behaviors and cultivating clearer communication, thereby offering crucial support for mutual aid activities.

Our findings, when viewed from a cultural capital perspective, highlight the significant role of cultural propaganda in both predicting and facilitating mutual aid behaviors. Parboteeah et al. identify positive and interactive social connections with others as an essential prerequisite for mutual aid behavior (78). The research undertaken by Martí offers additional backing for the notion that community cultural and educational activities cultivating collective values can stimulate collective action for societal good (79). Additionally, through publicity and educational activities, residents can gain awareness of the disasters prevalent in their community and recognize the limits of their own individual capacities. Therefore, they will realize the necessity of mutual aid. Finally, community-organized emergency activities offer opportunities for informal interaction; these opportunities help to lower interpersonal barriers common in modern communities and cultivate emotional connections among participants.

Another significant discovery from this study is the moderating effect of disaster experiences on the relationship between community disaster mitigation environments and individual emergency behaviors. Similarly, the research by Que. et al. corroborates the notion that a more severe disaster experience increases the likelihood of residents deciding to engage in mutual aid actions. Specifically, communities that have previously encountered disasters tend to implement more thorough disaster risk reduction measures to lessen the potential for future catastrophic losses; such measures include upgrading emergency facilities, conducting frequent disaster drills, and promoting the spread of disaster-related knowledge. Residents who have lived through disasters exhibit an increased awareness of potential risks (65). It motivates them to participate actively in these disaster reduction efforts, which increases the level of their emergency behaviors.

### 6.2 Policy implications

The analysis results of this study suggest several implications:

The first strategy involves establishing efficient, direct channels for information dissemination; it also includes implementing a standardized, formatted information delivery mechanism. During disaster events, it is possible for relevant departments to utilize apps, official websites, and social media platforms for the real-time dissemination of authoritative disaster warning information. Additionally, an improvement in residents’ literacy education regarding information can assist in developing their capacity for accurate information identification and filtering, thereby strengthening their sense of self-protection and emergency response capabilities.

A second area for consideration is the community’s capacity to enhance emergency culture and disaster preparedness. The organization of theme cultural activities, for instance community emergency assistance, enables the community to propagate concepts of helping and harmonizing; this can also cultivate “neighborhood mutual aid and social harmony” as individual values in daily life. Emergency culture elements can be integrated into community infrastructure development; for instance, setting up emergency knowledge propaganda boards or creating emergency evacuation route maps offers residents with subtle emergency culture education. Selecting and cultivating individuals who act as positive role models is another approach; through these examples, the community can inspire broader resident participation in collective emergency work. Therefore, the collective ability of the community in mutual assistance and response is enhanced by this effort.

A third key point involves enhancing residents’ emergency literacy, considering that individual emergency knowledge and skills form the decision-making basis for emergency behavior. Designing customized education and training programs is essential; these programs should be designed to residents’ age groups and occupational categories and must emphasize households as fundamental units of emergency preparedness. Encouragement should be offered for residents to sufficiently prepare prior to potential emergencies. To effectively improve residents’ response and collaborative abilities, organizing comprehensive exercises is also important. These exercises should focus on preemptive measures, relocation and avoidance, self-help, and mutual aid, and they should be coupled with targeted drills for common regional disasters.

## 7 Conclusion

This study, according to social cognition theory, explores the differential driving factors of emergency behavior, focusing on the subjective perspectives of community residents. Results from the data analysis report a key role for specific factors in promoting self-help emergency behavior among residents; these factors include individual emergency cognition (particularly emergency knowledge and skills) and the publicization of information about the community disaster mitigation environment. On the other hand, core explanatory elements for mutual aid emergency behavior have been identified as individual emergency cognition associated with emergency emotions and cultural propaganda in the community disaster mitigation environment. The test of the moderating effect demonstrates that disaster experience positively moderates the effect of the community disaster mitigation environment on emergency behavior.

This study has made valuable progress and yielded some notable results, which are crucial for developing a society focused on reducing disaster risks. However, this study nevertheless faces certain limitations. First, despite this study analyzing related variables based on an existing theoretical model, it is worth noting that factors influencing community emergency behavior are often multifaceted and complex, with many potential influencing variables yet to be explored further. Future research could employ methods such as in-depth interviews and experiments to delve deeper into additional influential variable factors and refine the existing theoretical models accordingly. Second, this study examines the intrinsic linkages among cognitive factors, environmental conditions, and emergency behavior from a residents’ perspective. However, it does not explore the relationship between cognitive factors and environmental conditions. Future research could broaden its scope by incorporating more pathways of interaction between these variables. Additionally, while control variables such as gender, age, and occupation were examined in this study, future research should also consider individual traits like proactive personality or achievement motivation, which may significantly influence residents’ proactive behavior. By including these factors, the study could provide a more comprehensive understanding of how cognitive, environmental, and individual traits interrelate in shaping emergency behaviors.

## Data Availability

The data analyzed in this study is subject to the following licenses/restrictions: The authors do not have permission to share data. Requests to access these datasets should be directed to Haocun Zhao, 11224718@cumt.edu.cn.
